# From Sedentary Time to Sedentary Patterns: Accelerometer Data Reduction Decisions in Youth

**DOI:** 10.1371/journal.pone.0111205

**Published:** 2014-11-04

**Authors:** Mai J. M. Chinapaw, Mark de Niet, Maïté Verloigne, Ilse De Bourdeaudhuij, Johannes Brug, Teatske M. Altenburg

**Affiliations:** 1 Department of Public and Occupational Health and the EM GO Institute for Health and Care Research, VU University Medical Center, Amsterdam, The Netherlands; 2 Science Support, Utrecht, The Netherlands; 3 Department of Movement and Sport Science, Ghent University, Ghent, Belgium; 4 Department of Epidemiology and Biostatistics and EMGO Institute for Health and Care Research, VU University Medical Center, Amsterdam, The Netherlands; University of Geneva, Switzerland

## Abstract

**Aim:**

This study aims to establish evidence-based accelerometer data reduction criteria to accurately assess total sedentary time and sedentary patterns in children.

**Methods:**

Participants (n = 1057 European children; 9–13 yrs) were invited to wear an accelerometer for at least 6 consecutive days. We explored 1) non-wear time criteria; 2) minimum daily valid wear time; 3) differences between weekday and weekend day; and 4) minimum number of days of accelerometer wear by comparing the effects of commonly used data reduction decisions on total sedentary time, and duration and number of prolonged sedentary bouts.

**Results:**

More than 60 consecutive minutes of zero counts was the optimal criterion for non-wear time. Increasing the definition of a valid day from 8 to 10 hours wear time hardly influenced the sedentary outcomes, while the sample size of children with more than 4 valid days increased from 69 to 81%. On weekdays, children had on average 1 hour more wear time, 50 minutes more total sedentary time, 26 minutes more sedentary time accumulated in bouts, and 1 more sedentary bout. At least 6 days of accelerometer data were needed to accurately represent weekly sedentary time and patterns.

**Conclusions:**

Based on our results we recommend 1) a minimum of 60 minutes of consecutive zeros as the most realistic criterion for non-wear time; and 2) including at least six days with minimum eight valid hours to characterize children's usual total sedentary time and patterns, preferably including one weekend day.

## Introduction

In recent years, interest in the potential adverse health effects of excessive sedentary time has grown. A recent review concluded that there is insufficient evidence for a prospective association between excessive sedentary and biomedical health indicators (such as body mass index, blood pressure and glucose levels) in children and adolescents, except for aerobic fitness [1]. One of the limitations of the studies included in this review was the validity and reliability of the measures of sedentary behaviour. Accurate and reliable measurement of sedentary behaviours is key for further studies on its health effects.

Recent studies in adults have shown that not only the total amount of sedentary time may be detrimental, but also the way it is accumulated [2]–[4]. Healy et al [5] found that a higher number of breaks in sedentary time was beneficially associated with waist circumference, body mass index, triglycerides, and 2-hour plasma glucose. This relationship was independent of total sedentary time, moderate-to-vigorous intensity activity time, and the average intensity of physical activity. Although in adults evidence on the adverse health effects of specifically prolonged sitting is accumulating, there is insufficient evidence in children [6]. Therefore, it is important to examine the impact of data reduction decisions on sedentary patterns in addition to total sedentary time to better enable further research into this issue. In the present study we define sedentary patterns as the way sedentary time is accumulated throughout the day (e.g. the number and duration of sedentary bouts).

Accelerometers are increasingly being used to objectively assess sedentary time in children and adolescents. Accelerometers cannot differentiate between sitting still and standing still and therefore provide an estimate of lack of movement, rather than sedentary time. However, they are widely used as an objective measure of sedentary time in the research literature. Despite only being assessed in few studies, accelerometers appear to provide a valid measure of sedentary time in youth [7], [8]. Ridgers et al. [8] compared ActiGraph cut-points for sedentary time, objectively-assessed periods of free-living sitting, and sitting plus standing time, using the activPAL, demonstrating that a cut-point of 100 counts per minute (cpm) reflects the time children spend sitting. A major advantage of accelerometry is that it not only provides an estimate of total sedentary time but also how it is accumulated (e.g. accumulated in bouts or intermittently throughout the day). Unfortunately, there is no consensus on accelerometer data reduction criteria, leading to considerable variation in data reduction procedures being used in different studies. A number of studies have shown the influence of different data reduction decisions on estimates of physical activity [9]–[11]. Few studies have examined the impact of different data reduction procedures on total sedentary time. Ojiambo et al [10] found that the number of days required to obtain 70, 80, and 90% reliability for total sedentary time was 4.7, 8.1, and 18.3, respectively. To the best of our knowledge, no previous studies have examined the influence of data reduction procedures on the number or duration of sedentary bouts.

The first decision in data reduction is the definition of non-wear time. This is usually defined by a period of consecutive zeros in accelerometer output. However, when the person is wearing the accelerometer, but sitting still and not moving, the accelerometer can also accumulate multiple consecutive zero counts. Thus, the definition of non-wear time may have a large impact on estimates of sedentary time. In the literature on child and adolescent studies, six definitions of ‘non-wear time’ are reported, ranging from 10–180 minutes of consecutive zero counts [12]. The next decision is the minimum number of valid hours per day required to characterize children's usual sedentary patterns. Frequently used is a minimum of 8 or 10 hours of valid hours per day to constitute a valid day [12]. Furthermore, it is important to know whether weekdays differ from weekend days and the minimum number of valid wear days required representing ‘usual’ activity. To obtain reliable estimates of physical activity in children and adolescents, a number of monitoring days ranging between 4 and 9 is recommended [11]. However, a recent review showed that only 35% of studies relied on a minimum of 4 valid days [12]. Since sedentary behaviour may be more or less variable between days than physical activity, the minimum number of monitoring days may be different for sedentary behaviour.

This study aims to establish evidence-based accelerometer data reduction criteria for assessing sedentary time and patterns in children. We not only examine total sedentary time in children, but also duration and number of prolonged sedentary bouts. The research questions are: 1) What is the most realistic criterion for non-wear time to characterize children's usual sedentary time and patterns? 2) How many hours per day are required to characterize children's usual sedentary time and patterns? 3) Is there a difference in weekday versus weekend days regarding sedentary time and patterns? 4) How many days of monitoring are required to characterize children's usual sedentary time and patterns?

## Subjects and Methods

### Ethics statement

Ethical approval was obtained from medical ethical review committees in all participating countries and/or regions: The Medical Ethics Committee of the Ghent University Hospital in Belgium; The Bioethics Committee of Harokopio University in Greece: The Scientific and Ethics Committee of Health Sciences Council in Hungary; The Medical Ethics Committee of the VU University Medical Center in The Netherlands; and the ethics committees of the participating Basel, Bern, Aargau and St. Gallen in Switzerland. Both parents provided written informed consent and all children gave verbal consent.

### Study design and sample

Data were obtained as part of the ENERGY (EuropeaN Energy balance Research to prevent excessive weight Gain among Youth) project (www.projectenergy.eu) [13], [14]. The sample for the current analyses consists of girls and boys from the five participating countries where accelerometer data were collected; Belgium, Greece, Hungary, Switzerland, and The Netherlands. Per country, three cities were selected with a different degree of urbanisation (low, middle, and high tertile). Schools were randomly selected in the three cities to reach a representative sample of children per country, aged between 9 and 13 years old. The data collection took place between March and September 2010. Accelerometer data were collected from approximately 200 children per country. The study design, selection criteria, and sample size are described in detail elsewhere [13], [15].

### Procedure

Participants were asked to wear an ActiGraph accelerometer (models GT1M, Actitrainer and GT3X) for at least 6 consecutive days during school-term time. The uniaxial output of these monitors is compatible [16]. A 15-second epoch was used to capture the rapid transitions in activities typical for children [17]. More importantly, the 15-second epoch is also used in the studies examining the validity of accelerometers for assessing sedentary time in youth [8], [18]. Each child was asked to wear the ActiGraph at all waking hours and remove the device only for water-based activities. The raw data were analysed using a customized software program developed by MC, TA and MdN in MATLAB. For inclusion in the current data analysis, each participant needed at least one day with a minimum of ten valid hours of wear time to have the same children in the analyses when comparing 8 versus 10 hours minimum wear time. We selected a cut-point of 100 cpm for sedentary behaviour [18], [19]. Different cut-points are used to define sedentary time. A recent review by Cain et al [12] found that child studies used nine sedentary cut points with the most common being (100 cpm). Fisher et al. [18] and Ridgers et al. [8] compared common accelerometer sedentary cut-points for children and concluded that the cut-point of <100 cpm is the most appropriate. Therefore, we decided to use this cut-point in the present study. We selected a cut-point of 3000 cpm [19] for moderate to vigorous intensity activity (MVPA).

Demographic data (age, gender, and ethnicity) were obtained by self-report [20]. We collected data on body height and weight according to standardized procedures [13]. The children were measured in light clothing without shoes. Body height was measured with a Seca Leicester Portable stadiometer (accuracy of 0.1 cm). Weight was measured with a calibrated electronic scale SECA 861 (accuracy of 0.1 kg). Body mass index (BMI) was calculated for each child, and weight status (normal weight, overweight, obesity) was based on the International Obesity Task Force criteria [21].

### Statistical Analyses

All analyses were done using MATLAB version R2009a and SPSS version 18.0. Our MATLAB program calculated total sedentary time (all minutes <100 counts), sedentary time accumulated in bouts of at least 10-minutes, number of sedentary bouts, average duration of sedentary bouts, number of breaks between sedentary bouts, and the average duration of breaks. A bout was defined as a period of at least ten consecutive minutes <100 counts. A break was considered as transition in accelerometer count from <100 cpm to ≥100 cpm in between two sedentary bouts.

To decide on the most realistic criterion for non-wear time, we calculated and compared the number of non-wear time periods per day defined by three different criteria: ≥20, ≥30 or ≥60 consecutive minutes of zero counts with no interruptions allowed (Step 1). These criteria were chosen based on common use in the literature [12]. Since it is not likely that children remove the accelerometer multiple times per day, we decided on the most realistic non-wear time criterion based on the maximum number of non-wear time periods per day and the influence on valid wear time per day.

Second, using the most realistic criterion decided in Step 1, we examined the difference in sedentary outcomes and sample size, with more than 4 valid days, using a minimum of 8 versus 10 hours valid wear time per day (Step 2). Third, potential differences between weekday and weekend sedentary outcomes were examined in the subgroup of children with at least one weekend day. Differences between 8 versus 10 hours of valid days, and between weekdays and weekend days, were judged based on their practical relevance (Step 3).

Finally, based on the decisions taken in Step 1 and 2, we assessed the minimum number of valid days required to characterize children's sedentary behaviour to achieve reliabilities of 0.70, 0.80, and 0.90, respectively, using the Spearman-Brown prophecy formula [22], which uses ICC as a measure of reliability as defined:
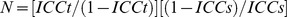



where N is the number of days needed, ICCt is the desired level of reliability (typically 0.7–0.9) and ICCs is the single-day reliability based on seven valid days. Therefore, this last analysis was performed in a subsample of children with at least 7 valid days.

## Results

Table 1 shows the characteristics of the study sample. Children's mean age was 11.7 years old. In boys 26% were overweight and 5% obese. In girls, 20% were overweight and 4% obese. The mean cpm was 536 in boys and 449 in girls.

**Table 1:** Sample characteristics (n = 1047).

	Boys (n = 506)	Girls (n = 531)
Mean age (yrs ± SD)	11.7±0.8	11.6±0.8
Mean BMI (kg/m^2^ ± SD)	19.1±3.5	18.8±3.3
Overweight	26%	20%
Obese	5%	4%
Mean counts per minute (± SD)	536±146	449±130
ethnicity (% native)	86%	85%

Table 2 presents the number of non-wear periods, as well as the valid wear time per day, using the three different criteria for non-wear time. The maximum number of non-wear periods ranged from four in the 60 minutes consecutive zero definition of non-wear time, to six in 30 minutes, and ten in 20 minutes of consecutive zeros. The number of children with four or more non-wear periods was 224 for the 20 minutes consecutive zero definition, 74 for the 30 minutes consecutive zero definition, and none for the 60 minutes consecutive zero definition of non-wear time. Since it seems unrealistic that children take off the accelerometer 4 or more times a day, we chose 60 minutes consecutive zeros as the optimal cut point for non-wear time. Valid wear time varied slightly between the different cut points between 13.2 and 13.6 hours per day.

**Table 2:** Number of non-wear periods and valid wear time per day defined by three different criteria in European school children (n = 1057).

	≥20 min zero counts	≥30 min zero counts	≥60 min zero counts
Median # non-wear periods per valid day (range)	3.1 (1–10)	2.7 (1–7)	2.1 (1–4)
Valid wear time (hrs/day)	13.2	13.4	13.6

Table 3 shows that increasing the definition for a valid day, from 8 to 10 hours wear time, hardly influenced the sedentary outcomes. Since the sample size of children with more than 4 valid days increased from 69 to 81%, we decided that at least 8 hours valid wear time is optimal to estimate sedentary time or patterns in children.

**Table 3:** Sedentary behaviour outcomes in European school children comparing 8 versus 10 hrs wear time as valid day (n = 1057)^a^.

	8 hrs wear time (range)	10 hrs wear time (range)
# valid days	5.7 (1–13)	5.1 (1–12)
% children >4 valid days	81%	69%
Median total sedentary time (min/day)(range)	492 (319–797)	507 (312–797)
Median (± SD) sedentary time accumulated in 10 min-bouts (min/day)(range)	123 (16–545)	123 (4–545)
Median # of sedentary bouts of at least 10 min (range)	6.8 (1–25)	7.0 (0.3–25)
Median sedentary bout duration (min)(range)	18 (10–155)	18 (10–145)
Median duration of breaks (min)(range)	80 (0.3–376)	81 (0–376)
Median duration of MVPA breaks (min)(range)	3.6 (0–37)	3.7 (0–37)

a non-wear time defined as ≥60 min consecutive zero counts.

Table 4 shows the difference in sedentary outcomes on weekdays and weekend days in the subsample with both valid week and weekend day data. On weekdays, children had on average 1 hour more valid wear time, 51 minutes more total sedentary time, 26 minutes more sedentary time accumulated in bouts of at least 10-minutes, 1.2 more sedentary bouts, and 11 minutes more MVPA. Therefore, we recommend including one weekend day to obtain an adequate estimate of children's usual time or sedentary patterns.

**Table 4:** Sedentary behaviour outcomes in European school children on week and weekend days (Median)^a^^,^ b.

	Weekday (range)	Weekend day (range)
# children with valid days (N)	1053	872
Valid hours (N = 869^b^) (range)	13 (9–17)	12 (8–17)
Total sedentary time (min/day) (N = 869^b^) (range)	506 (305–753)	455 (174–855)
Sedentary time accumulated in 10 min-bouts (min/day) (N = 857^b^) (range)	127 (13–428)	101 (0–529)
# of sedentary bouts per day (N = 857^b^) (range)	7.2 (1–18)	6.0 (1–25)
Sedentary bout duration (min) (N = 875^b^) (range)	18 (7–31)	17 (5–15)
Duration of breaks (min) (N = 828^b^) (range)	87 (63–124)	83 (51–122)
MVPA (min/day) (N = 869^b^) (range)	36 (25–49)	25 (13–40)

a non-wear time defined as ≥60 min consecutive zero counts and at least 8 hours wear time per day.

b calculated for the sample with both valid week and weekend days.

Table 5 shows the reliability coefficients of sedentary behaviour variables over several days of monitoring. Single-day ICC was 0.29 for total sedentary time, 0.30 for sedentary time accumulated in 10-min bouts and 0.37 for number of 10-min sedentary bouts. The number of days required to obtain 80% reliability for total sedentary time, sedentary time accumulated in 10-min bouts and number of 10-min sedentary bouts was ten, nine and seven days, respectively. The number of days required to obtain 70% reliability for total sedentary time, sedentary time accumulated in 10-min bouts and number of 10-min sedentary bouts was 6, 5 and 4 days, respectively. We recommend including at least 6 valid days to reliably characterize children's usual sedentary time and patterns.

**Table 5:** Reliability of sedentary behaviour outcomes over several days of measurement in European school children (n = 269)^a^.

Outcome	ICC^b^	Days of measurement^c^		
		R = 0.7	R = 0.8	R = 0.9
Total sedentary time	0.29	6	10	22
Sedentary time accumulated in 10 min-bouts	0.30	5	9	21
# of sedentary bouts of at least 10 min	0.37	4	7	15

a non-wear time defined as ≥60 min consecutive zero counts and at least 8 hours wear time per day.

b On the basis of 7 days of monitoring including at least 1 weekend day.

c Predicted by Spearman–Brown Prophecy formula.

## Discussion

The present study provides evidence for accelerometer data reduction criteria for assessing sedentary time and patterns in children. Based on our results we recommend 1) a minimum of 60 minutes of consecutive zeros as the most realistic criterion for non-wear time; and 2) including at least six days with minimum eight valid hours to characterize children's usual sedentary time and patterns, preferably including one weekend day. Based on these data reduction criteria, 647 of 1057 children (64%) provided valid accelerometer data.

Our results on number of non-wear periods for 20, 30 and 60 minutes of consecutive zeros (as the definition of non-wear time) are in line with the study of Toftager et al. [23], in a slightly older sample of Danish adolescents. Our findings regarding differences in sedentary patterns between weekday and weekend are similar with findings from studies examining physical activity [10], [11]. In comparison with physical activity studies, ICCs seem comparable [10] or somewhat higher [11] for physical activity, and therefore recommended monitoring days for physical activity vary between studies.

An important advantage of accelerometers is that they cannot only measure the duration of total sedentary time but also the way it is accumulated. Although rarely used, this information is of utmost importance for unravelling the potential adverse health effects of sedentary behaviour. Raw accelerometer counts are unit-less and dimension-less, and thus require calibration in order to be translated in a biologically meaningful way. Unfortunately, there is no consensus on data reduction criteria and as a result criteria vary widely between studies, [12] complicating the comparability between studies. Previous studies used different data reduction decisions leading to different sedentary behaviour outcomes, and therefore limited comparability between studies. It is important to examine the influence of different data reduction criteria on accelerometer-based estimates of sedentary time and patterns, and reach consensus regarding the optimal accelerometer data reduction protocol. Moreover, the description of data reduction criteria is often not detailed enough to enable reproduction. The present study provides evidence for such a consensus. We highly recommend uniform use and clear description of data reduction criteria in future studies.

Our study is the first to examine the influence of different data reduction criteria on accelerometer-based estimates of sedentary time and patterns. One recent study examining the influence of data reduction decisions on physical activity included total sedentary time, [10] but not sedentary bouts or breaks. The authors concluded that a minimum of 7–9 days of monitoring including at least 1 weekend day was required to achieve 80% reliability in their slightly younger cohort of 86 children aged between 4 and 10 years (mean age 7±2 years). However, their definition of a valid day was a minimum duration of 6 hours per day compared to 8 hours in our study. Further, they defined periods of 20 minutes or more consecutive zero counts as non-wear time, versus 60 minutes or more in our study.

Only a few studies have examined sedentary bouts and breaks in children using slightly different definitions. We defined a sedentary bout as a minimum of 10 consecutive minutes below the cut-point of 100 cpm without tolerance. The hypothesis underlying the adverse health effects of uninterrupted sitting is that prolonged lack of muscle contractions leads to suppression of skeletal muscle lipoprotein lipase (LPL) activity (a protein important for controlling plasma triglyceride catabolism, HDL cholesterol, and other metabolic risk factors) [24]. Since any movement resulting in counts above the cut-point of 100 cpm is caused by muscle contractions, we allowed no tolerance during a sedentary bout (i.e., no counts ≥100). This is in contrast to Colley et al., who defined a sedentary bout as at least 20 minutes with ≥80% of minutes below the 100 cpm cut-point (e.g., 16 out of 20 minutes or 32 out of 40 minutes) [25]. The bout stopped when <80% was below the 100 cpm cut-point or when there were ≥3 consecutive minutes ≥100 cpm or any observations ≥1500 cpm (cut-point for moderate intensity). Carson and Janssen defined a sedentary bout of at least 30 minutes with ≥80% of minutes below the 100 counts cut-point, with a maximum of 5 consecutive minutes ≥100 counts [26].

There is no consensus on the definition of a break in sedentary time. Our definition of a break is a transition in accelerometer count from <100 cpm to ≥100 cpm in between two sedentary bouts. The first study on breaks in sedentary time was by Healy et al. in Australian adults. In this study, a break was defined as any time of at least one minute in which the accelerometer count reached from <100 cpm to ≥100 cpm [5]. Thus, their definition of breaks equals the number of light, moderate, and vigorous activity periods, irrespective of whether the sedentary time was accumulated in bouts. Three studies in children defined a sedentary break. Colley et al. defined a break as ≥3 consecutive minutes ≥100 cpm or each minute ≥1500 cpm (i.e., a cut-point for moderate intensity) [25]. Carson & Janssen [26] defined a break as each 15-second epoch ≥100 cpm within a sedentary bout of at least 30 minutes with a maximum of 5 consecutive minutes zeros. Harrington et al [27] defined a break as each 15-second period ≥100 cpm, thereby reflecting time spent on light, moderate, and vigorous physical activity. These different definitions lead to large differences in the prevalence of sedentary bouts and breaks. Thus, consensus on the optimal definition of sedentary bouts and breaks is urgently needed, not only for the comparability between studies, but also to accumulate evidence on potential adverse health effects of different sedentary patterns.

Strengths of this study are the large, multi-country dataset, the uniform data collection across countries, and the stepwise approach to reach optimal data reduction criteria for characterizing usual sedentary time and patterns in school children. Moreover, this is the first study examining the influence of data reduction criteria on sedentary bouts and breaks in sedentary time, in addition to total sedentary time. A limitation of our study is that our sample includes children aged 9–13 years; thus, findings may not be generalizable to younger or older age groups. As mentioned previously, accelerometers are no gold standard for measuring sitting, since they cannot distinguish between sitting still and standing still. This limitation of accelerometers needs also to be kept in mind when examining health effects of sedentary behaviour, since both standing and lying may have different health effects. However, since accelerometers are frequently used to objectively assess sedentary time in children, standardized data reduction criteria are urgently needed. The Spearman-Brown prophecy analyses were performed in a smaller sample of children with at least seven valid days. This sample had somewhat lower total sedentary time (16 minutes less on weekdays and 119 minutes less on weekend days), as well as number of sedentary bouts (6 versus 5 bouts on weekdays and 6.6 versus 7.3 bouts on weekend days). Therefore, we recommend analysis to confirm these findings in other larger samples. A final limitation is that we decided on the most realistic non-wear time criterion based on an arbitrary maximum number of non-wear time periods per day. However, confirmation of the adequacy of our decision regarding non-wear time criteria in a laboratory study with video observation may not provide the same data as collected in real-life circumstances.

We strongly recommend the development of an evidence-based accelerometer data reduction protocol for assessing sedentary time and patterns in children. Accurate measurement of sedentary time and patterns is essential for future research into the potential adverse health effects of sedentary behaviour in children. Future studies should not only examine total sedentary time, but also how sedentary time is accumulated to better understand what the minimum prolonged sedentary time is leading to adverse health effects, as well as the duration and intensity of breaks attenuating this effect. The above-mentioned hypothesis that the lack of muscle contractions induced by sitting suppresses skeletal muscle LPL activity, contributing to increased cardiometabolic risk, implies that reducing the duration of sedentary bouts is key. Therefore, we believe that future epidemiological studies should focus on examining the potential health effects of the duration of sedentary *bouts* rather than breaks.

In conclusion, we recommend that future accelerometer data reduction protocols include at least 6 days with minimum 8 hours valid wear time, and preferably one weekend day, in order to represent usual sedentary time and patterns in school children aged 9–13 yrs. Further, a definition of non-wear time of ≥60 minutes of consecutive studies seems optimal.
